# Associations between perceived organizational support, workplace violence, and employee outcomes: a cross-sectional study

**DOI:** 10.3389/fpsyg.2026.1853679

**Published:** 2026-08-24

**Authors:** Saud A. Alsulaiman, Fatimah Albrekkan

**Affiliations:** 1 Department of Mass Communication, College of Humanities and Social Sciences, King Saud University, Riyadh, Saudi Arabia; 2 Department of Psychiatry, College of Medicine, King Saud University, Riyadh, Saudi Arabia

**Keywords:** employee outcomes, organizational support theory, perceived organizational support, Saudi Arabia, workplace violence

## Abstract

**Introduction:**

Workplace safety is a fundamental organizational priority that ensures employees’ psychological well-being for sustainable performance and productivity. Organizational support is crucial for tackling workplace violence and fosters employee and organizational outcomes.

**Methods:**

This cross-sectional study aimed to measure perceived organizational support and its association with workplace violence and employee outcomes using an online survey of 558 employees from 17 organizations in Saudi Arabia.

**Results:**

The findings showed that perceived organizational support was negatively associated with workplace violence, such as gender discrimination and perceived sexual harassment, and positively associated with employee outcomes, such as job satisfaction and intention to leave a job.

**Discussion:**

This study shows the value of organizational support in mitigating workplace violence across sectors and provides practical strategies to foster a healthy, supportive work environment.

## Introduction

Workplace safety protects employees and supports their well-being. A positive safety culture backed by organizational support is critical for organizational success. Workplace violence (WPV) can hamper organizational efforts to provide a safe and supportive work environment for employees. A United Nations report stated that more than 20% of employees worldwide have experienced some form of physical, sexual, or psychological violence and harassment (United Nations, 2022).

Numerous studies have shown that organizations showing their support for employee well-being through perceived organizational support (POS) demonstrate higher levels of job retention, performance, and satisfaction (Eisenberger et al., 1986; Riggle et al., 2009; Kurtessis et al., 2017; Rockstuhl et al., 2020). This study therefore aimed to examine the relationship between POS and WPV, specifically perceived sexual harassment (PSH) and perceived gender discrimination (PGD), as well as employee outcomes such as intention to leave (INTL), job stress (JS), and job satisfaction (JSAT), among employees in the private and public sectors in Saudi Arabia. To the best of the researchers’ knowledge, research on this topic is scarce, as limited studies in Saudi Arabia have investigated these phenomena simultaneously at a large number of organizations, particularly the associations between POS and WPV, such as PGD and PSH, as well as the association between POS and employee outcomes, highlighting the importance of this study. In addition, most studies examining WPV in Saudi Arabia have focused on health workers; this study is therefore one of the first empirically grounded investigations of POS and WPV among employees from various sectors in Saudi Arabia. It also attempts to understand whether workplace policies and initiatives regarding WPV are associated with higher POS.

This study provides a better understanding of the relationship between POS, WPV, and employee outcomes, helping organizations identify shortcomings and create healthy and safe workplace environments. The findings will also help organizations comprehend the value of POS for employees’ well-being in Saudi Arabia.

## Literature review

According to Eisenberger et al. (1986), perceived organizational support “reflects the global beliefs concerning the extent to which the organization values employees’ contributions and cares about their well-being” (p. 500). Research has shown that employees who are valued and appreciated at work have positive attitudes and behaviors toward their organizations (Rhoades and Eisenberger, 2002). Other studies have shown how POS can increase work engagement and protect psychological well-being (Wang, 2024). The importance of this is reflected in findings that WPV can hinder organizations’ efforts to support their employees. Numerous studies have shown that employees with higher PGD and PSH experience higher JS and INTL and lower JSAT (Fitzgerald et al., 1988; Foley et al., 2005; Merkin and Shah, 2014; Triana et al., 2019). Having proper policies to prevent WPV and protect employees’ safety and well-being is one of the crucial forms of organizational support associated with employees’ happiness and satisfaction, lowering stress and their intention to leave their job (Aytaç and Dursun, 2012; Chang et al., 2018; Dai et al., 2025).

## Theoretical framework

### Organizational support theory (OST)

This theory was first introduced in 1986 by Eisenberger et al. (1986), and assumes that employees often personify the organization and perceive their experiences, such as those with supervisors and HR policies, as emanating from the organization itself. Hence, employees form general beliefs about their organizations based on their own experiences, including whether organizations value them and care about their well-being. Employees’ beliefs are shaped in various ways such as fair treatment, supervisor support, working conditions, rewards, and recognition (Eisenberger et al., 1986). The theory also assumes that organizational support helps satisfy employees’ emotional needs, including a sense of belonging, approval, and self-esteem. This in turn leads to the norm of reciprocity proposed by Gouldner (1960); he argued that the norm of reciprocity exists in all cultures, and that in organizational contexts, employees feel obligated to respond to organizational support through work commitment, job satisfaction, and reduced absenteeism (Gouldner, 1960; Eisenberger et al., 1986). The OST theory is based on the Social Exchange Theory proposed by Peter Blau in 1964, which assumes that relationships inside organizations are based on reciprocal exchange: when employees feel they have high organizational support, they feel morally obligated to return this favor and reciprocate through work dedication and commitment (Blau, 1964). Rhoades and Eisenberger’s meta-analysis of POS in 2002 found that it was associated with benefits for employees such as fair treatment, support from supervisors, rewards and recognition, access to training, and good work conditions (Rhoades and Eisenberger, 2002). The study also found that these benefits resulted in affective commitment, in which employees feel attached to and loyal to their organizations, and that having benefits led to greater job satisfaction and a better mood among employees. Such benefits help employees work diligently and avoid absenteeism. A study conducted among 1,111 employees from three organizations in France found that POS was associated with higher work engagement, greater feelings of work meaning, and lower job stress (Canboy et al., 2023).

Conversely, a study conducted among 1,804 nurses in emergency departments from 13 cities in China found that higher WPV was associated with lower POS. Nurses who experienced WPV reported lower POS, and that POS was associated with resilience and perceived professional identity (Tao et al., 2025). Another study conducted among 1,761 nurses in China found that higher WPV was associated with higher INTL, and POS was associated with lower WPV and INTL (Liu et al., 2018). Huang et al. (2024) found that POS among 825 nurses was associated with resilience, and that self-efficacy mediated the relationship between POS and resilience. Another study among school teachers in Australia found that POS works as a barrier counter to the negative consequences of WPV, such as bullying, as POS moderates the relationship between WPV and INTL (Djurkovic et al., 2008). A large study in South Africa involving almost 14,000 employees across various economic sectors found that WPV, specifically bullying by co-workers and supervisors, was associated with INTL, and suggested that POS was negatively associated with INTL (Van Schalkwyk et al., 2011). Another study conducted across two samples in 2016 and 2019 of more than 4,000 teachers and educators from pre-K to grade 12 in Canada found that POS was negatively associated with bullying and served as a buffering factor that reduced job stressors and gender discrimination (Harlos et al., 2023).

While the literature has shown the value of POS in tackling WPV, the World Health Organization (WHO) has stated that the issue of the underreporting of WPV, such as verbal abuse, threats, and harassment, is common worldwide. This is due to several reasons, including lack of organizational support, employees’ perceptions that these incidents are necessary components of the job, and fear of retaliation (World Health Organization, 2026). Similarly, numerous meta-analyses have found that underreporting WPV is a common issue across many organizations because of the absence of clear policies, lack of organizational support, the nature of reporting mechanisms, and fear of retaliation (Lanctôt and Guay, 2014; Spencer et al., 2023; Xu et al., 2025; Abreu et al., 2026).

These findings highlight the importance of increasing employee awareness, implementing clear policies, and improving reporting systems. Although extensive research has examined the association between POS and WPV worldwide, limited research has investigated this phenomenon, especially in Saudi Arabia. Most previous studies conducted in Saudi Arabia have examined only WPV and its relationship with employee and workplace outcomes. For instance, Alwabili et al.’s (2024) study, conducted among 239 psychiatrists, suggested that WPV was highly prevalent and resulted in increased stress and lower work productivity. Another study conducted among 324 healthcare workers suggested that almost 50% of them had experienced at least one type of WPV and that verbal abuse was the most prevalent form of WPV. This study also found that WPV was associated with higher JS among healthcare workers (Harthi et al., 2020). Another study across more than 1,054 home healthcare workers similarly found that more than 67% of the sample experienced at least one form of WPV, and that higher WPV incidence was associated with nurses and younger workers (Al-Sagheir et al., 2021).

Therefore, drawing from the literature review, this study aimed to:

- Assess the level of POS among employees in Saudi Arabia, and
- Examine the relationships between POS and WPV, and the associated employee outcomes.

We also hypothesized that:

- POS is negatively associated with WPV, particularly PGD and PSH,
- POS is positively associated with JSAT,
- POS is negatively associated with JS, INTL, and
- The existence of WPV policies and awareness campaigns is associated with higher POS.

## Methods

### Study design, setting, and participants

A cross-sectional online study was conducted using non-probability purposive sampling among employees working in the public and private sectors across Saudi Arabia as part of a larger research project (Albrekkan et al., 2026). The inclusion criteria included individuals aged 18 and above who were working in the private or public sectors in Saudi Arabia, including banks, hospitals, universities, and ministries. Exclusion criteria of the study were individuals younger than 18 years and those with communication barriers. We used the Raosoft® online sample size calculator with a 95% confidence level, a 5% margin of error, and a response distribution of 50% to determine the sample size. Based on the target population, the estimation was 384 participants (The Global Economy, 2026). The study occurred between November 2023 and March 2025. A formal invitation letter, including the survey link, was sent to 20 governmental, semi-governmental, and private organizations across Saudi Arabia, requesting their participation and the distribution of the questionnaire to all their employees. The data collection process was lengthy as it required acquiring approvals from all participating organizations to coordinate and distribute the survey. Only three organizations, however, declined to participate without providing a specific explanation, making the total 17 organizations. Of the 898 participants who started the study, 558 completed the study, yielding a survey completion rate of around 62%. We only included completed surveys in the final analysis. Also, the unavailability of the number of employees who received the survey invitation prevented us from calculating the response rate (Albrekkan et al., 2026).

### Study instruments

This study used validated mainstream measurement tools to assess POS and WPV, including PGD, PSH, and employee outcomes, such as JSAT, JS, and INTL, as part of a larger research project (Albrekkan et al., 2026). All elements used a five-point Likert scale (1 = strongly disagree to 5 = strongly agree), with higher scores indicating greater agreement on each scale. All scales were checked for content validity by three experts, and modifications were made based on the study’s objectives. All WPV and employee outcomes scales were checked for construct validity using exploratory factor analysis (EFA) with Principal Axis Factoring (PAF) and Principal Component Analysis (PCA) with Parallel Analysis and Closeness-to-unidimensionality (MI-REAL, ECV, UniCo) to assess dimensionality, internal structure, and validity (Albrekkan et al., 2026). All scales demonstrated good factorability (UniCO > 0.91, ECV > 0.90, and MI-REAL ranged between 0.144 and 0.229) (Albrekkan et al., 2026). We then ran exploratory factor analysis of the 13 POS items using principal component analysis (PCA) with Direct Oblimin rotation and eigenvalues greater than 1. The Kaiser–Meyer–Olkin (KMO) value was 0.94, and Bartlett’s test of sphericity was χ^2^(78) = 5096.16, *p* < 0.001, with one factor explaining 59% of the variance. The reliability of POS was checked using Cronbach’s alpha, and it was 0.94. The reliability of WPV and outcome employee scales was assessed using Cronbach’s alpha, and all scales showed excellent reliability (Cronbach’s *α* > 0.89) (Albrekkan et al., 2026).

The study instrument consisted of seven sections: a sociodemographic questionnaire developed by the researchers, Perceived Organizational Support Questionnaire (POS), Perceived Gender Discrimination Questionnaire (PGD), Perceived Sexual Harassment Questionnaire (PSH), Job Stress Questionnaire (JS), Job Satisfaction Questionnaire (JSAT), and Intention to Leave Questionnaire (INTL). The developed questionnaire assessed sociodemographic and employment-related information. It included questions about age, sex, marital status, level of education, and employment type.

The adapted POS questionnaire was composed of thirteen 5-point Likert items (1 = strongly disagree to 5 = strongly agree) based on Eisenberger et al. to measure perceived organizational support (Eisenberger et al., 1986; Muyidi, 2020; Muyidi et al., 2023). Higher scores signaled greater perceived organizational support in the workplace.

The PGD questionnaire was composed of eight items adapted from Sanchez and Brock’s scale to assess perceptions of gender discrimination in the workplace (Sanchez and Brock, 1996; Muyidi, 2020; Muyidi et al., 2023). Participants were asked to rate the extent to which they agreed with statements reflecting discriminatory attitudes, behaviors, and institutional practices related to gender in their workplace. Responses were recorded on a 5-point Likert scale ranging from 1 (strongly disagree) to 5 (strongly agree), with higher scores indicating greater perceived gender discrimination.

The PSH questionnaire was composed of sixteen items from the widely used Sexual Experiences Questionnaire (SEQ), measuring sexual harassment in the workplace. The scale comprises three components: unwanted sexual attention, gender harassment, and sexual coercion. Participants indicated how often they experienced each type of harassment from employees, co-workers, and supervisors using a Likert scale from 1 (never) to 5 (very often) (Fitzgerald et al., 1988; Muyidi, 2020; Muyidi et al., 2023).

The JS questionnaire was composed of six items based on Motowidlo et al., measuring job stress on a five-point Likert scale (1 = strongly disagree, 5 = strongly agree) (Motowidlo et al., 1986; Muyidi, 2020; Muyidi et al., 2023). Higher scores indicate greater levels of job stress.

Five items from Brayfield and Rothe’s job satisfaction scale were used to assess JSAT (1 = strongly disagree, 5 = strongly agree), with higher scores meaning greater agreement with the statements (Brayfield and Rothe, 1951; Muyidi, 2020; Muyidi et al., 2023).

The INTL instrument was composed of four items used to assess intentions to step down from their jobs (1 = strongly disagree, 5 = strongly agree), adapted from Mobley et al.’s model of turnover intention among employees, particularly in private organizations (Mobley et al., 1978; Muyidi, 2020; Muyidi et al., 2023). Higher scores indicated employees’ higher intention to leave their jobs.

### Data analyses

We used IBM SPSS Statistics Version 29 for statistical analysis. Descriptive analyses included frequencies, means, standard deviations, and Cronbach’s *α*, and inferential analyses included multivariable generalized linear gamma regression and multivariable linear regression. Multivariable generalized linear models with a gamma distribution and log link were performed to examine whether associations between POS, PSH, PGD, and employee outcomes, such as INTL, JS, and JSAT, exist. Multivariable linear regression was used to examine the associations between independent variables, like POS, and dependent variables (employee outcomes), such as JSAT and INTL. Regression coefficients (*β*), *p*-values, and 95% confidence intervals indicated the strength and significance of the relationships between variables. Prior to interpretation, model assumptions were assessed before the analyses. Normality tests were performed using skewness, kurtosis, histograms, and the Shapiro–Wilk test, which indicated that some variables were positively skewed. The Box-Whisker plots were used to assess outlier values within the distributions of the metric variables across categorical variable levels. Collinearity and multicollinearity among the tested variables were assessed using the Tolerance (Ti) and Variance Inflation (VIF) indices. Therefore, the gamma distribution with a log link was used, which is robust to non-normal outcome distributions through the specification of a gamma family and a log link (Albrekkan et al., 2026).

## Results

### Sample characteristics

The demographic characteristics, such as sex, age, and level of education, are presented in Table 1 (Albrekkan et al., 2026).

**Table 1:** Sociodemographic characteristics of the study participants (*N* = 558) adapted from Albrekkan et al. (2026).

Sociodemographic characteristics	Frequency	Percentage
Sex		
Male	216	38.7
Female	342	61.3
Age group		
18–24 years old	19	3.4
25–34 years old	210	37.6
35–44 years old	208	37.3
45–54 years old	86	15.4
≥55 years old	35	6.3
Marital status		
Single	207	37.1
Married	308	55.2
Divorced or widowed	43	7.7
Educational level		
High school	20	3.6
Diploma	27	4.8
Bachelor’s degree	294	52.7
Master’s degree	117	21
Doctorate (Ph.D., M.D.)	100	17.9
Employment type		
Full-time	539	96.6
Part-time	11	2
Others	8	1.4

### Level of POS, WPV, and employee outcomes

The POS overall mean score was (M = 45.03, SD = 11.94). The highest-rated POS items reflected formal equality and protection mechanisms, particularly policies supporting the recruitment of both men and women (M = 3.88, SD = 1.12), followed by ensuring equal rights in income and work hours (M = 3.78, SD = 1.18), the existence of policies aimed at reducing workplace harassment (M = 3.64, SD = 1.13), and the existence of policies and laws encouraging employees to treat each other with respect (M = 3.63, SD = 1.14). In contrast, the lowest-rated indicators concerned outcomes related to employee experience, including policies perceived to enhance overall job satisfaction (M = 3.08, SD = 1.24) and provide the best job opportunities based on qualifications (M = 3.10, SD = 1.27). The overall mean scores for PSH, PGD, and employee outcomes are presented in Table 2 (Albrekkan et al., 2026).

**Table 2:** Means and standard deviations of variables adapted from Albrekkan et al. (2026).

Scale	Mean	Standard deviation
POS	45.03	11.94
PGD	19.63	7.97
PSH	18.48	5.70
JSAT	19.20	6.46
JS	19.16	6.45
INTL	13.63	5.68

### WPV policies and employees’ experience

Almost 50% of employees (*n* = 264) reported that their organizations had a workplace policy, compared with 17% (*n* = 94) who said they did not, and 36% (*n* = 200) who said they did not know. Moreover, 48% of employees (*n* = 268) said that their organizations had not conducted any awareness campaigns about WPV, compared with 30% who said they had (*n* = 169) and 22% who did not know (*n* = 121). The majority of employees (85%) said they had never reported any WPV in their current organizations (*n* = 474), compared to 15% who had done so (*n* = 84).

### Associations between POS, WPV experience, WPV policies, and awareness campaigns

Table 3 shows the results of a multivariable generalized linear model with a gamma distribution and log link examining factors associated with the mean POS score. Adjusted risk ratios (RRs) with 95% confidence intervals (CIs) were reported. After adjusting for all covariates in the model, POS was not significantly associated with age or gender. Tables 4 and 5 present the multivariable linear regression of the association between POS and employees’ job satisfaction and intention to leave.

**Table 3:** Multivariable generalized linear gamma regression analysis of mean perceived organizational support (POS) scores.

Predictors	Multivariable adjusted risk rate	95% CI for RR		*p*-value
		Lower	Upper	
(Intercept)	55.136	47.700	63.731	<0.001
Sex = Male	1.010	0.973	1.048	0.609
Age group	0.988	0.970	1.007	0.210
Does your organization have a policy regarding workplace violence? (Yes/No)	1.092	1.051	1.135	<0.001
Has your organization conducted an awareness communication campaign about workplace violence? (Yes/No)	1.083	1.038	1.129	<0.001
Are you aware of anyone in your organization who has faced workplace violence? Yes vs. No	0.973	0.901	0.974	<0.001
Has your organization conducted an awareness communication campaign about workplace violence? Yes vs. No	1.083	1.038	1.129	<0.001

Dependent variable: perceived organizational support (POS) score. Estimation method: Maximum likelihood with log-link, model fit indexes (AIC = 4050.8, BIC = 4202.2).

**Table 4:** Multivariable generalized linear gamma regression analysis of employees’ mean perceived sexual harassment (PSH) score.

Predictors	Multivariable adjusted risk rate	95% CI for RR		*p*-value
		Lower	Upper	
(Intercept)	14.735	12.414	17.489	<0.001
Have you ever reported any workplace violence in your current organization? Yes *Vs* No	0.944	0.899	0.992	0.022
Are you aware of anyone in your organization who has faced workplace violence? Yes *Vs* No	1.050	1.011	1.091	0.011
Has your organization conducted an awareness communication campaign about workplace violence? Yes vs. No	0.990	0.950	1.031	0.622
Does your organization have a policy regarding workplace violence? Yes vs. No	1.028	0.989	1.068	0.157
Mean perceived organizational support (POS) score	0.998	0.996	1.000	0.029

Dependent variable: Overall mean perceived sexual harassment (SEQ) score. Estimation method: Maximum likelihood with log-link, model fit indexes (AIC = 3010.3, BIC = 3239.5).

**Table 5:** A multivariable linear regression analysis of the employees’ mean perceived job satisfaction (JSAT) score.

Predictors	Unstandardized Beta coefficient	95% CI for Beta coefficient		*p*-value
		Lower	Upper	
(Constant)	19.503	15.598	23.408	<0.001
Does your organization have a policy regarding workplace violence? Yes vs. No	−0.119	−0.903	0.665	0.765
Are you aware of anyone in your organization who has faced workplace violence? Yes vs. No	0.466	−0.392	1.324	0.287
Mean perceived organizational support (POS) score	0.102	0.056	0.148	<0.001
Mean perceived gender discrimination (PGD) score	0.346	−0.163	0.855	0.182
Mean overall perceived sexual harassment (PSH) score	0.034	−0.035	0.103	0.335
Mean perceived job stress (JS) score	−0.158	−0.234	−0.082	<0.001
Mean perceived intention to leave job (INTL) score	−0.617	−0.703	−0.531	<0.001
Have you ever reported any workplace violence in your current organization? Yes vs. No	1.196	0.085	2.308	0.035

Dependent variable: Employees’ mean perceived job satisfaction (JS) score. Model overall significance: *f*(16,541) = 44.4, *p*-value <0.001. Model *R*-squared = 0.568, adjusted *R*-squared = 0.555.

The findings showed that participants working in organizations with a workplace violence policy reported significantly higher POS scores than those without such a policy (RR = 1.09, 95% CI [1.05, 1.14], *p* < 0.001). This corresponded to an estimated 9% increase in the average POS score among those with workplace policies. Similarly, the results showed that the presence of workplace violence awareness communication campaigns was associated with a higher POS (RR = 1.08, 95% CI [1.04, 1.13], *p* < 0.001), resulting in an approximately 8% increase in the average POS for those with awareness campaigns. However, being aware of someone in the organization who had faced workplace violence was significantly associated with a lower POS score (RR = 0.94, 95% CI [0.90, 0.97], *p* = 0.001), resulting in a 6% decrease in the average POS score. The results also showed that personally reporting an incident of workplace violence was associated with a lower POS (RR = 0.94, 95% CI [0.90, 0.99], *p* = 0.028) (see Table 3).

### Associations between POS, PGD, and PSH

The findings showed that POS was significantly associated with PGD. A gamma regression model with a log link was fitted; the model fit was assessed using AIC and BIC (AIC = 3543.5, BIC = 3593.7). The findings showed that higher POS scores were significantly associated with lower PGD scores (RR = 0.98, 95% CI [0.982, 0.987], *p* < 0.001), indicating that each one-unit increase in POS was associated with an estimated 2% decrease in the mean PGD score.

Moreover, POS was significantly associated with PSH. A gamma regression model with log link was fitted; a model fit was assessed using AIC (3010.3) and BIC (3239.5). The findings showed POS was associated with PSH (RR = 0.998, 95% CI [0.996, 1.000], *p* = 0.029), indicating that each one-unit increase in POS was associated with an estimated 1% decrease in the mean PSH score (see Table 4).

### Associations between POS and employee outcomes

The multivariable linear regression was also conducted to examine the association between POS and JSAT, adjusting for all variables (Table 5). The model was significant (*f*(16,541) = 44.4, *p* < 0.001), explaining 55% of the variance (*R*^2^ = 0.568, adjusted *R*^2^ = 0.555). POS was positively associated with JSAT (*β* = 0.102, 95% CI: −0.056 to 0.148, *p* = 0.001), indicating that higher POS resulted in higher JSAT among employees.

Furthermore, the multivariable linear regression test was conducted to examine the association between POS and INTL, adjusting for covariates (Table 6). The model was significant (*f*(13,544) = 63.76, *p* < 0.001), explaining 59% of the variance (*R*^2^ = 0.604, adjusted *R*^2^ = 0.594). POS was negatively associated with INTL (*β* = −0.058, 95% CI: −0.096 to −0.019, *p* = 0.003), indicating that higher POS was associated with lower INTL.

**Table 6:** Multivariable linear regression analysis of the employees’ intention to leave the job (ITNL) score.

	Unstandardized Beta coefficient	95% CI for Beta coefficient		*p*-value
		Lower	Upper	
(Constant)	19.547	16.479	22.614	<0.001
Does your organization have a policy regarding workplace violence? Yes vs. No or Do not know	0.315	−0.341	0.971	0.346
Are you aware of anyone in your organization who has faced workplace violence? Yes vs. No	0.736	0.052	1.420	0.035
Mean perceived organizational support (POS) score	−0.058	−0.096	−0.019	0.003
Mean perceived gender discrimination (PGD) score	0.410	−0.014	0.834	0.058
Mean overall perceived sexual harassment (PSH) score	−0.020	−0.078	0.037	0.487
Mean perceived job stress (JS) score	0.200	0.137	0.262	<0.001
Mean perceived job satisfaction (JSAT) score	−0.436	−0.496	−0.376	<0.001

Dependent variable: Employees’ mean perceived intention to leave job score. Model overall significance: *f*(13,544) = 63.76, *p*-value <0.001. Model *R*-squared = 0.604, adjusted *R*-squared = 0.594.

## Discussion

This study is conceptually ambitious as it is one of the first in Saudi Arabia to examine the relationship between POS and WPV, such as PGD and PSH, and employee outcomes, such as JS, INTL, and JSAT. As detailed earlier in the literature review, most studies done in Saudi Arabia have focused on WPV and employee outcomes in healthcare settings (Harthi et al., 2020; Al-Sagheir et al., 2021; Alwabili et al., 2024). A recent systematic review and meta-analysis of WPV studies conducted between 2013 and 2023 among nurses in Saudi Arabia showed that more than 60% experienced WPV, with deleterious effects on their health and well-being. It was found that WPV had negatively associated with nurses’ mental health, increased job stress, and reduced job performance, satisfaction, and productivity, which in turn affected the quality of care (Abdelmalik et al., 2025).

Studies worldwide have also shown that WPV has a significant association with employees’ health and psychological well-being and is significantly associated with increased job stress, intention to leave, and lower job satisfaction (Fitzgerald et al., 1988; Sofield and Salmond, 2003; Foley et al., 2005; Merkin and Shah, 2014; Triana et al., 2019). For instance, a recent study reviewed 36 WPV studies conducted in India and found that various forms of WPV, such as verbal abuse and physical violence, were prevalent and high among healthcare workers, especially in urgent units, emergency departments, and psychiatric wards (Saha et al., 2026). The study found that WPV had negative consequences for victims, including social and psychological issues, ranging from job dissatisfaction, intention to quit a job, sleep and memory issues to suicide attempts. The study suggested several approaches to tackle WPV and ensure safety in health settings, including improving security measures and healthcare infrastructure, such as common areas, lighting, and cleanliness (Saha et al., 2026). At the same time, POS provides tremendous value to organizations by helping minimize the damage associated with WPV. Extensive studies worldwide have shown that POS can act as a buffer, countering the negative relationship with WPV (Djurkovic et al., 2008; Harlos et al., 2023), warranting further investigation into the potential moderating role of POS in WPV and employee outcomes, especially in Saudi Arabia. Research on this topic in Saudi Arabia remains scarce. Therefore, based on organizational support theory (OST), this study serves as a foundational contribution to the limited literature on POS and its associations with WPV and employee outcomes in Saudi Arabia.

Our study showed that POS is significantly associated with a decreased level of gender discrimination and sexual harassment in the workplace, highlighting the potential value of POS in minimizing sexual and gender discrimination in the workplace. This corresponds with the findings of Harlos et al. (2023), which found that POS plays a key role as a buffer mitigating job stressors and sexual and gender discrimination. Although our study found that POS is associated with decreased PGD and PSH levels, the magnitude of the effect is relatively small, and its practical importance may be meaningful when considered across a large workforce. Furthermore, Triana et al. (2019) conducted a comprehensive meta-analysis of the effects of gender discrimination on employees and found that it was significantly associated with negative outcomes, including job attitudes and behaviors such as satisfaction, commitment, absenteeism, involvement, and intention to leave. They also found that gender discrimination was significantly correlated with negative psychological health, such as increased stress, depression, and anxiety, and contributed significantly to employees’ mental health. The study also showed that gender discrimination can physically harm employees’ health, causing fatigue, headaches, and other health problems.

In addition, our study found that POS was associated with higher JSAT and lower INTL among employees. This aligns with the assumptions of OST and is consistent with numerous studies indicating that POS increases JSAT and reduces absenteeism among employees (Eisenberger et al., 1986; Riggle et al., 2009; Kurtessis et al., 2017; Rockstuhl et al., 2020). Nevertheless, we did not find a significant relationship between POS and JS.

Workplace safety is governed by well-established international and national standards and regulations that protect employees from workplace violence and ensure a safe working environment. According to the Occupational Safety and Health Administration (OSHA) General Duty Clause [Section 5(a)(1)], employers must provide a workplace “free from recognized hazards” leading to serious harm or death. This includes systematic hazard identification and control through risk assessments and the application of a hierarchy of controls (Occupational Safety and Health Administration (OSHA), n.d.; National Institute for Occupational Safety and Health, 2015); providing adequate training to workers on hazards, safe procedures, and reporting mechanisms; maintaining logs of work-related injuries or illnesses; and emergency preparedness plans. Chapter 8 of Saudi Arabia’s Labor Law establishes a comprehensive national framework for workplace safety, highlighting the employer’s legal duty to ensure a safe working environment and protect employees from occupational hazards. This represents a mandatory organizational obligation, requiring employers to develop and enforce workplace safety regulations aligned with national standards to safeguard workers’ health and well-being (Saudi Labor Law, 2005; Saudi Labor Law, n.d.).

Indeed, our findings show that organizational policies, initiatives, and awareness campaigns on WPV are significantly associated with higher POS among employees, which corresponds with the literature and the assumptions of OST. This mirrors the findings of an extensive study conducted in the United States, which found that organizational support, including clear policies, safety protocols, and WPV prevention programs, is associated with higher POS among employees who feel that their organizations care about their health and well-being (Landsbergis et al., 2018). The results also coincide with a substantial body of research demonstrating that lack of organizational support and the absence of comprehensive workplace policies hinder employees’ ability to find an appropriate channel to report WPV (Alwabili et al., 2024; Elsharkawy et al., 2025; Gholam and El-Fatah, 2025). A recent study of 400 healthcare workers in the western region of Saudi Arabia suggested that a lack of awareness of reporting systems, the absence of clear organizational procedures and support, and perceptions that reporting is ineffective were some of the reasons that prevented healthcare workers from reporting incidents of WPV (Gholam and El-Fatah, 2025).

Accordingly, many organizations worldwide understand the importance of a safe and supportive workplace environment that enhances employees’ health and well-being, and of fostering reciprocal relationships in which employees are more dedicated and committed to their organizations. A Gallup report found that engaged employees are vitally important, both to the success of organizations and for improving employees’ own well-being, and that employees’ engagement can substantially contribute to increased productivity, higher satisfaction, better performance, and profitability, as well as reduced absenteeism and turnover (Gallup, 2026). This explains the growing number of organizations focusing on employees’ health and well-being. For instance, Johnson & Johnson launched several initiatives that focused on enhancing employees’ health and well-being, such as the Wellness 360 initiative, which fosters employees’ and their families’ mental health and provides access to educational tools such as webinars and daily messages that help employees manage stress, depression, and eating disorders (Shapiro, 2018). Another example is Amazon, which maintains clear workplace policies, such as its “Code of Business Conduct Ethics,” which promotes ethical behaviors and equal opportunities for employees. Amazon’s policies prohibit harassment and discrimination and encourage employees to report violations of its code of conduct (Amazon Inc., 2024). Starbucks created a panoramic program that emphasizes the importance of a safe workplace environment. It aims to equip employees with proper training, including how to respond to active shooters, de-escalation techniques, and maintaining safe stores. Starbucks also maintains clear policies and guidelines for addressing WPV (Starbucks Coffee Company, 2026). Such programs, conducted across many organizations worldwide, indicate the value and importance of creating a safe and supported workplace environment that offers employees competitive benefits and career development, thereby enhancing their organizational performance.

### Limitation

Our study has some limitations. The use of a cross-sectional study and purposeful non-probability sampling (an online survey) may have introduced selection bias as participants were not randomly selected. This also limits the generalizability of the findings and does not permit causal inference. Future studies could also use other methods, such as mixed methods research by utilizing simple random sampling and include interviews or focus groups to gain in-depth insights into the topics under study. It might also be useful, if available, to obtain data and records from organizations regarding WPV incidents, policies, and employee outcomes to assess the status quo of employees in Saudi Arabia. Another limitation is that the response rate and non-response bias could not be assessed because the total number of employees who received the survey invitation was unknown to the researchers in this study. However, we were only able to provide the completion rate of the study.

## Conclusion

Workplace safety is a mandatory obligation for all organizations. Ensuring a safe workplace environment is pivotal not only for employees’ well-being but also for organizational financial stability and employee retention. Unlike previous studies that focused solely on WPV and its relation to employee outcomes in healthcare settings in Saudi Arabia, this study provides a clear assessment of POS in relation to WPV, policies, and employee outcomes among employees in Saudi Arabia in a wide variety of settings, helping organizations understand the value of POS in maintaining a safe and healthy workplace environment.

## Data Availability

The raw data supporting the conclusions of this article will be made available by the authors, without undue reservation.
